# The Importance of Continuous Glucose Monitoring-derived Metrics Beyond HbA1c for Optimal Individualized Glycemic Control

**DOI:** 10.1210/clinem/dgac459

**Published:** 2022-07-31

**Authors:** Hidenori Yoshii, Tomoya Mita, Naoto Katakami, Yosuke Okada, Takeshi Osonoi, Katsumi Aso, Akira Kurozumi, Satomi Wakasugi, Fumiya Sato, Ryota Ishii, Masahiko Gosho, Iichiro Shimomura, Hirotaka Watada

**Affiliations:** Department of Medicine, Diabetology & Endocrinology, Juntendo Tokyo Koto Geriatric Medical Center, Koto-ku, Tokyo 136-0075, Japan; Department of Metabolism & Endocrinology, Juntendo University Graduate School of Medicine, Tokyo, 113-8421, Japan; Department of Metabolic Medicine, Osaka University Graduate School of Medicine, Osaka, 565-0871, Japan; First Department of Internal Medicine, School of Medicine, University of Occupational and Environmental Health, Japan, Kitakyushu 807-8555, Japan; Nakakinen Clinic, Ibaraki 311-0113, Japan; Aso Clinic, Shizuoka, Japan 410-0041, Japan; First Department of Internal Medicine, School of Medicine, University of Occupational and Environmental Health, Japan, Kitakyushu 807-8555, Japan; Department of Metabolism & Endocrinology, Juntendo University Graduate School of Medicine, Tokyo, 113-8421, Japan; Department of Metabolism & Endocrinology, Juntendo University Graduate School of Medicine, Tokyo, 113-8421, Japan; Department of Biostatistics, Faculty of Medicine, University of Tsukuba, Ibaraki 305-8575, Japan; Department of Biostatistics, Faculty of Medicine, University of Tsukuba, Ibaraki 305-8575, Japan; Department of Metabolic Medicine, Osaka University Graduate School of Medicine, Osaka, 565-0871, Japan; Department of Metabolism & Endocrinology, Juntendo University Graduate School of Medicine, Tokyo, 113-8421, Japan

**Keywords:** glucose variability, continuous glucose monitoring, individualization of glycemic control, time in range

## Abstract

**Context:**

Current guidelines recommend assessing glycemic control using continuous glucose monitoring (CGM) and hemoglobin A1c (HbA1c) measurement.

**Objective:**

This study aimed to clarify the characteristics of patients who might benefit from CGM metrics in addition to HbA1c monitoring.

**Methods:**

CGM metrics, specifically time in range (TIR), time below range (TBR), and time above range (TAR), were determined in 999 outpatients with type 2 diabetes and compared between HbA1c categories (HbA1c < 53 mmol/mol [7.0%, HbA1c <  _53_], HbA1c 53-63 mmol/mol [7.0-7.9%, HbA1c _53-63_], HbA1c 64-74 mmol/mol [8.0-8.9%, HbA1c _64-74_], and HbA1c ≥ 75 mmol/mol [9.0%, HbA1c ≥  _75_]) and between patients with identical HbA1c categories who were stratified by age, types of antidiabetic agents, and renal function.

**Results:**

For HbA1c <  _53_ category, patients aged ≥ 65 years had a significantly higher nocturnal TBR than those aged < 65 years. For HbA1c <  _53_ and HbA1c _53-63_ categories, patients receiving insulin and/or sulfonylureas had a significantly higher TAR and TBR, and a lower TIR than those not receiving these drugs, and for HbA1c _64-74_ category, they had a significantly higher TBR. For HbA1c <  _53_, HbA1c _53-63_, and HbA1c _64-74_ categories, patients with an estimated glomerular filtration rate (eGFR) < 60 mL/min/1.73 m^2^ had a significantly higher TBR during some periods than those with an eGFR ≥ 60.

**Conclusion:**

Higher HbA1c levels do not always protect against hypoglycemic episodes. Our data demonstrate that using CGM metrics to complement HbA1c monitoring is beneficial, especially in older people, users of insulin and/or sulfonylureas, and patients with chronic kidney disease.

The growing prevalence of diabetes has become a significant global challenge to the health and quality of life of individuals and family members, and it also negatively affects societies (1). The main cause of diabetic complications is damage to tissues and organs caused by persistent hyperglycemia (2); the development and progression of these complications are therefore best prevented by optimal glycemic control. In this regard, early and tight glycemic control has been shown to reduce the risk of microvascular complications more than less intensive control (3-7).

Based on these data, current guidelines developed by the American Diabetes Association (ADA) and the Japan Diabetes Society (JDS) have set a hemoglobin A1c (HbA1c) control target of less than 53 mmol/mol (7.0%) for optimal diabetes management (6, 8, 9). However, the ADA guidelines specify that HbA1c has a number of limitations that should be taken into account in clinical practice (10). For instance, previous observational studies demonstrated that both overly low and high HbA1c levels were associated with higher mortality in patients with type 2 diabetes mellitus (T2DM) (11, 12). The underlying mechanisms by which low and high HbA1c are associated with increased mortality may differ considerably. Although high HbA1c is associated with increased mortality caused by diabetes-related complications and cancer (3), the cause of increased mortality in T2DM patients with low HbA1c remains to be clarified. In this regard, HbA1c reflects average glucose levels over the previous few months, but it provides no information on glucose variability or hypoglycemia. It has been suggested that glucose variability and hypoglycemia may contribute to increased mortality. In addition, numerous factors such as age, race, anemia, and chronic kidney disease (CKD) may have a major impact on HbA1c measurement (10). These limitations of HbA1c underscore the need for complementary methods to assess glucose levels.

Continuous glucose monitoring (CGM) has emerged as an optimal method to obtain a comprehensive glycemic profile, including data on glucose variability and hypoglycemia. In fact, the ADA guidelines recommend assessing glycemic control not just with HbA1c measurement, but also with CGM based on time in range (TIR), defined as the percentage of the time spent within the target glucose range. TIR is a useful metric of glycemic control and glucose patterns, and it is closely associated with HbA1c and the risk of microvascular complications (10). Previous studies reported that a TIR of 70% corresponded to an estimated HbA1c of 7.0% in patients with type 1 diabetes (13) or 6.7% in patients with type 1 diabetes or T2DM (14). Based on these data, the ADA currently recommends a TIR target > 70% with a time below range (TBR) < 3.9 mmol/L target of < 4% and a TBR < 3.0 mmol/L target of < 1%, in parallel with an HbA1c target < 53 mmol/mol (10). These new metrics could help improve clinical management by providing more information than HbA1c.

However, considering the cost and effort necessary to assess CGM data, it is important to determine the characteristics of patients who are likely to derive clinical benefit from the use of CGM metrics that complement HbA1c monitoring. This exploratory cross-sectional study aimed to address this issue from the point of view of detecting glucose variability and hypoglycemia.

## Materials and Methods

### Study Design

This study is an exploratory subanalysis of an ongoing observational, prospective cohort study that aims to investigate the relationships between glucose fluctuations evaluated with CGM and the incidence of composite cardiovascular events over a 5-year follow-up period (15). This study used the baseline data from the cohort study. This study has been registered in the University Hospital Medical Information Network Clinical Trials Registry, which is a nonprofit organization in Japan that meets the requirements of the International Committee of Medical Journal Editors (UMIN000032325).

### Study Population

The study population consists of Japanese patients with T2DM who regularly attend the outpatient diabetes clinics of 34 institutions across Japan. The study design, inclusion criteria, and exclusion criteria were published previously (15). Briefly, outpatients aged ≥ 30 years and ≤ 80 years with stable diabetes control were included. Patients with a history of cardiovascular events were excluded.

Consecutive subjects were screened. Patients who met the eligibility criteria were asked to participate in the present study. A total of 1000 patients who met the eligibility criteria were recruited between May 2018 and March 2019. One patient withdrew consent. The protocol was approved by the institutional review board of each participating institution in compliance with the Declaration of Helsinki and current legal regulations in Japan. Written informed consent was obtained from all participants after a full explanation of the study.

### Biochemical Tests

Blood samples were obtained at visits after overnight fasting. Renal function tests, lipid levels, and HbA1c (National Glycohemoglobin Standardization Program) were measured with standard techniques. Urinary albumin excretion was measured by a latex agglutination assay using a spot urine sample. The estimated glomerular filtration rate (eGFR) was calculated using a previously defined formula (16).

### Diabetic Retinopathy and Diabetic Nephropathy Assessment

The presence of diabetic retinopathy (DR) was determined by trained ophthalmologists. The patients were grouped into 2 groups based on medical records: no DR, or DR categorized as simple DR, preproliferative DR, or proliferative DR. Diabetic nephropathy (DN) was defined according to the urinary albumin excretion level: < 30 mg/g creatinine was defined as normoalbuminuria, 30 to 300 mg/g creatinine was defined as microalbuminuria, and ≥ 300 mg/g creatinine was defined as macroalbuminuria.

### CGM With the FreeStyle Libre Pro Device

The FreeStyle Libre Pro (FLP) (Abbott Japan, Tokyo, Japan) CGM (FLP-CGM) device, which measures glucose levels every 15 minutes for up to 14 days, was used as previously reported (15, 17-19). Other than wearing the FLP-CGM, there were no restrictions on participants’ daily lives. Downloaded data sets were further analyzed. Glucose variability was assessed based on the mean amplitude of glycemic excursion (MAGE) (20), SD, and glucose coefficient of variation (CV). MAGE was calculated as the arithmetic mean of the differences between consecutive peaks and nadirs, provided that the differences were greater than 1 SD of the mean glucose value. CV (%) was calculated by dividing SD by the mean of the corresponding glucose readings. TIR was defined as the percentage of time spent in the target range between 3.9 and 10.0 mmol/L (TIR^3.9-10 mmol/L^), above the target range (TAR^ > 10 mmol/L^, TAR^ > 13.9 mmol/L^), or below the target range (TBR^ < 3.9 mmol/L^, TBR^ < 3.0 mmol/L^). Because a previous study demonstrated that FLP-CGM was less accurate during the first 24 hours after insertion (from the first day to the second day) and during the last 4 days of its 14-day lifetime (21), we analyzed FLP-CGM data over the middle 8-day period. Daytime was defined as the period between 6:00 am and midnight, and nighttime was defined as the period between midnight and 6:00 am. In addition, we examined what percentage of patients failed to fulfill the criterion of having a TBR^ < 3.9 mmol/L < ^4% or a TBR^ < 3.0 mmol/L^ < 1% (10).

### Data Analysis

To investigate whether FLP-CGM–derived metrics differed according to HbA1c, patients were divided into 4 HbA1c categories: HbA1c < 53 mmol/mol (7.0%, HbA1c <  _53_), HbA1c 53-63 mmol/mol (7.0-7.9%, HbA1c _53-63_), HbA1c 64-74 mmol/mol (8.0-8.9%, HbA1c _64-74_), and HbA1c ≥ 75 mmol/mol (9.0%, HbA1c ≥  _75_).

Next, patients were further classified into eight subgroups based on HbA1c and possible risk factors for glucose variability, specifically age (<65 years or ≥ 65 years), types of antidiabetic agents (insulin and/or sulfonylureas, no insulin or sulfonylureas), and renal function (eGFR < 60 mL/min/1.73 m^2^ or ≥ 60 mL/min/1.73 m^2^). Finally, we investigated the differences in FLP-CGM–derived metrics between patients in the same HbA1c category within each subgroup.

### Statistical Analysis

Results are presented as mean ± SD or median (first quartile [Q1], third quartile [Q3]) for continuous variables, and as number (proportion) of patients for categorical variables. For continuous variables, differences among the 4 HbA1c categories were tested with 1-way analysis of variance for normally distributed parameters or with the Kruskal-Wallis test for nonnormally distributed parameters, using the post hoc Tukey test or Steel-Dwass test as appropriate. Differences between patients stratified by age, types of antidiabetic agents, and renal function within each HbA1c category were tested with Student *t* test or Wilcoxon rank-sum test. For categorical variables, group comparisons were performed using Fisher exact test. All statistical tests were 2-sided with a 5% significance level. All analyses were performed using SAS software version 9.4 (SAS Institute, Cary, NC).

## Results

### Overall Patient Characteristics

The baseline clinical characteristics of the 999 patients with T2DM are summarized in Table 1. The number of patients per HbA1c category were as follows: HbA1c <  _53_, 500; HbA1c _53-63_, 386; HbA1c _64-74_, 85; and HbA1c ≥  _75_, 28. Patients with higher HbA1c levels were more likely to be younger and to have a higher body mass index (BMI), longer duration of T2DM, higher triglyceride levels, and higher eGFR. Also, patients with higher HbA1c levels were more likely to be treated with insulin and oral antidiabetic drugs, especially sodium-glucose cotransporter 2 (SGLT-2) inhibitors and glucagon-like peptide-1 (GLP-1) receptor agonists.

**Table 1.** Clinical characteristics of the whole patient population

Parameter	HbA1c < _53_ (n = 500)	HbA1c _53-63_ (n = 386)	HbA1c _64-74_ (n = 85)	HbA1c ≥ _75_ (n = 28)	*P* value
Age (y)	64.8 ± 9.1	65.2 ± 9.8	62.6 ± 10.1	58.3 ± 11.8	<0.001
Male (%)	318 (63.6)	223 (57.8)	51 (60.0)	16 (57.1)	0.338
Body mass index (kg/m^2^)	24.3 ± 3.8	24.6 ± 3.8	25.3 ± 4.1	26.9 ± 4.5	0.001
Estimated duration of diabetes (y)	10.9 ± 7.8	14.6 ± 8.6	15.6 ± 8.8	16.2 ± 9.6	<0.001
Systolic blood pressure (mmHg)	131.5 ± 15.3	131.3 ± 14.3	130.6 ± 15.2	126.5 ± 14.1	0.357
Diastolic blood pressure (mmHg)	76.1 ± 11.0	75.2 ± 11.0	73.8 ± 11.4	77.0 ± 11.2	0.244
HbA1c (%)	6.5 ± 0.3	7.3 ± 0.3	8.3 ± 0.3	9.8 ± 1.0	<0.001
HbA1c (mmol/mol)	47.3 ± 3.6	56.7 ± 2.8	67.6 ± 2.8	83.3 ± 10.6	<0.001
Total cholesterol (mmol/L)	4.79 ± 0.77	4.80 ± 0.82	4.85 ± 0.88	5.03 ± 1.26	0.515
LDL cholesterol (mmol/L)	2.65 ± 0.66	2.68 ± 0.69	2.68 ± 0.66	2.71 ± 1.08	0.943
HDL cholesterol (mmol/L)	1.59 ± 0.41	1.55 ± 0.39	1.48 ± 0.43	1.45 ± 0.44	0.052
Triglycerides (mmol/L)	1.07 (0.75-1.50)	1.15 (0.84-1.59)	1.22 (0.94-1.61)	1.69 (1.22-3.01)	<0.001
Estimated glomerular filtration rate (mL/min/1.73 m^2^)	73.0 ± 18.3	73.1 ± 20.6	72.3 ± 21.8	88.5 ± 42.5	0.001
Use of oral glucose-lowering agents (%)	426 (85.2)	360 (93.3)	80 (94.1)	28 (100.0)	<0.001
Metformin (%)	256 (51.2)	218 (56.5)	51 (60.0)	18 (64.3)	0.182
Sulfonylureas (%)	44 (8.8)	68 (17.6)	11 (12.9)	4 (14.3)	0.001
Glinides (%)	28 (5.6)	22 (5.7)	14 (16.5)	4 (14.3)	0.002
Dipeptidyl peptidase-4 inhibitors (%)	273 (54.6)	243 (63.0)	42 (49.4)	19 (67.9)	0.019
Sodium-glucose cotransporter 2 inhibitors (%)	91 (18.2)	106 (27.5)	24 (28.2)	10 (35.7)	0.002
Thiazolidinediones (%)	56 (11.2)	68 (17.6)	16 (18.8)	3 (10.7)	0.026
α-glucosidase inhibitors (%)	81 (16.2)	75 (19.4)	10 (11.8)	6 (21.4)	0.274
Glucagon-like peptide-1 receptor agonists (%)	17 (3.4)	37 (9.6)	15 (17.6)	5 (17.9)	<0.001
Insulin (%)	37 (7.4)	70 (18.1)	36 (42.4)	15 (53.6)	<0.001
Use of anti-hypertensive drugs (%)	249 (49.8)	181 (46.9)	37 (43.5)	16 (57.1)	0.493
Use of lipid-lowering agents (%)	295 (59.0)	227 (59.1)	51 (60.0)	22 (78.6)	0.225

Data are mean ± SD, median (Q1-Q3), or number of patients (%). Continuous data were compared using analysis of variance or the Kruskal-Wallis test as appropriate. Categorical data were compared using Fisher exact test.

Abbreviations: HDL, high-density lipoprotein; LDL, low-density lipoprotein.

The distribution of time spent in different blood glucose ranges across the 4 HbA1c categories is shown in Table 2. Increases in HbA1c were accompanied by progressively decreasing TIR and increasing TAR during an entire 24-hour period as well as during daytime and nighttime. The TBR^ < 3.9 mmol/L^ in the HbA1c <  _53_ category was significantly higher than in the other HbA1c categories. The TBR^ < 3.0 mmol/L^ in the HbA1c <  _53_ category was significantly higher than in the HbA1c _53-63_ and HbA1c ≥  _75_ categories during a full 24-hour period. That patients in the HbA1c ≥  _75_ category had a much higher TAR and lower TIR than those in the other HbA1c categories suggests that their glycemic control was poor and that they had few hypoglycemic episodes. Regarding other FLP-CGM–derived metrics, average glucose levels, SD, and MAGE increased progressively with increasing HbA1c (Table 3).

**Table 2.** TIR, TBR, and TAR of the whole patient population

Parameter	HbA1c < _53_ (n = 500)	HbA1c _53-63_ (n = 386)	HbA1c _64-74_ (n = 85)	HbA1c ≥ _75_ (n = 28)	*P* value
TIR^3.9–10 mmol/L^ (%)	90.5 (84.0-94.9)	78.1 (66.7-86.6)^*a*^	55.0 (37.0-67.6)^*a*^^,^^*b*^	27.2 (7.2-44.4)^*a*^^,^^*b*^^,^^*c*^	<0.001
TBR^ < 3.9 mmol/L^ (%)	0.65 (0.00-2.87)	0.00 (0.00-0.91)^*a*^	0.00 (0.00-0.78)^*a*^	0.00 (0.00-0.00)^*a*^^,^^*b*^	<0.001
TBR^ < 3.0 mmol/L^ (%)	0.00 (0.00-0.13)	0.00 (0.00-0.00)^*a*^	0.00 (0.00-0.00)	0.00 (0.00-0.00)^*a*^	<0.001
TAR^ > 10.0 mmol/L^ (%)	5.79 (2.47-12.6)	20.1 (11.5-31.8)^*a*^	44.1 (28.3-62.8)^*a*^^,^^*b*^	72.7 (55.6-92.8)^*a*^^,^^*b*^^,^^*c*^	<0.001
TAR^ > 13.9 mmol/L^ (%)	0.00 (0.00-0.52)	1.17 (0.00-4.17)^*a*^	8.98 (3.91-16.8)^*a*^^,^^*b*^	23.7 (14.4-56.2)^*a*^^,^^*b*^^,^^*c*^	<0.001
TIR^3.9–10 mmol/L^ (%), day	90.0(81.8-94.4)	74.5 (60.2-84.6)^*a*^	46.4 (36.7-63.9)^*a*^^,^^*b*^	25.3 (7.12-39.1)^*a*^^,^^*b*^^,^^*c*^	<0.001
TBR^ < 3.9 mmol/L^ (%), day	0.35 (0.00-2.43)	0.00 (0.00-0.52)^*a*^	0.00 (0.00-0.17)^*a*^	0.00 (0.00-0.00)^*a*^^,^^*b*^	<0.001
TBR^ < 3.0 mmol/L^ (%), day	0.00 (0.00-0.00)	0.00 (0.00-0.00)	0.00 (0.00-0.00)	0.00 (0.00-0.00)	0.026
TAR^ > 10.0 mmol/L^ (%), day	7.29 (3.13-16.2)	24.1 (14.1-39.4)^*a*^	53.3 (35.4-62.6)^*a*^^,^^*b*^	74.7 (60.9-92.9)^*a*^^,^^*b*^^,^^*c*^	<0.001
TAR^ > 13.9 mmol/L^ (%), day	0.00 (0.00-0.69)	1.39 (0.00-5.38)^*a*^	11.1 (4.49-21.4)^*a*^^,^^*b*^	25.6 (14.6-58.0)^*a*^^,^^*b*^^,^^*c*^	<0.001
TIR^3.9–10 mmol/L^ (%), night	97.4 (91.1-100.0)	94.0 (82.8-99.0)^*a*^	74.0 (46.9-89.6)^*a*^^,^^*b*^	29.7 (9.11-72.1)^*a*^^,^^*b*^^,^^*c*^	<0.001
TBR^ < 3.9 mmol/L^ (%), night	0.00 (0.00-4.69)	0.00 (0.00-1.04)^*a*^	0.00 (0.00-1.04)^*a*^	0.00 (0.00-0.00)^*a*^^,^^*b*^^,^^*c*^	<0.001
TBR^ < 3.0 mmol/L^ (%), night	0.00 (0.00-0.00)	0.00 (0.00-0.00)^*a*^	0.00 (0.00-0.00)	0.00 (0.00-0.00)	0.005
TAR^ > 10.0 mmol/L^ (%), night	0.00 (0.00-1.56)	3.13 (0.00-12.5)^*a*^	18.75 (6.25-53.1)^*a*^^,^^*b*^	70.31 (27.86-90.89)^*a*^^,^^*b*^^,^^*c*^	<0.001
TAR^ > 13.9 mmol/L^ (%), night	0.00 (0.00-0.00)	0.00 (0.00-0.00)^*a*^	0.52 (0.00-6.77)^*a*^^,^^*b*^	16.7 (2.60-47.4)^*a*^^,^^*b*^^,^^*c*^	<0.001

Data are median (Q1-Q3). Differences between the 4 HbA1c groups were tested using the Kruskal-Wallis test with the post hoc Steel-Dwass test.

Abbreviations: TAR, time above range; TBR, time below range; TIR, time in range.

^*a*^ *P* < 0.05 vs. HbA1c <  _53_.

^*b*^ *P* < 0.05 vs HbA1c _53-63_.

^*c*^ *P* < 0.05 vs HbA1c _64-74_.

**Table 3.** FLP-CGM-derived-metrics during a 24-hour period

Parameter	Subgroup	HbA1c _< 53_	HbA1c _53-63_	HbA1c _64-74_	HbA1c _≥ 75_	*P* value
Whole patient population						
Average glucose (mmol/L)		6.84 ± 1.01	8.22 ± 1.29^*a*^	9.92 ± 1.75^*a*^^,^^*b*^	12.58 ± 3.08^*a*^^,^^*b*^^,^^*c*^	<0.001
SD (mmol/L)		1.78 ± 0.48	2.17 ± 0.56^*a*^	2.69 ± 0.71^*a*^^,^^*b*^	2.88 ± 0.78^*a*^^,^^*b*^	<0.001
CV (%)		26.0 ± 5.7	26.4 ± 5.6	27.6 ± 6.9	23.4 ± 5.1^*b*^^,^^*c*^	0.005
MAGE (mmol/L)		4.77 ± 1.59	5.83 ± 1.95^*a*^	7.12 ± 2.34^*a*^^,^^*b*^	7.54 ± 2.56^*a*^^,^^*b*^	<0.001
Age	Years					
	<65 (n = 434)	n = 222	n = 151	n = 43	n = 18	
	≥65 (n = 565)	n = 278	n = 235	n = 42	n = 10	
Average glucose (mmol/L)	<65	6.79 ± 0.96	8.14 ± 1.20^*a*^	9.99 ± 1.83^*a*^^,^^*b*^	12.68 ± 3.04^*a*^^,^^*b*^^,^^*c*^	<0.001
	≥65	6.88 ± 1.05	8.28 ± 1.34^*a*^	9.84 ± 1.69^*a*^^,^^*b*^	12.39 ± 3.32^*a*^^,^^*b*^^,^^*c*^	<0.001
SD (mmol/L)	<65	1.72 ± 0.46^*d*^	2.12 ± 0.52^*a*^	2.56 ± 0.54^*a*^^,^^*b*^	2.85 ± 0.80^*a*^^,^^*b*^	<0.001
	≥65	1.83 ± 0.49	2.20 ± 0.59^*a*^	2.82 ± 0.84^*a*^^,^^*b*^	2.95 ± 0.76^*a*^^,^^*b*^	<0.001
CV (%)	<65	25.2 ± 5.3^*d*^	26.1 ± 5.3	26.3 ± 6.4	22.6 ± 3.8^*b*^	0.036
	≥65	26.5 ± 5.9	26.7 ± 5.8	28.8 ± 7.3	24.7 ± 6.9	0.087
MAGE (mmol/L)	<65	4.61 ± 1.49	5.80 ± 1.88^*a*^	6.80 ± 1.78^*a*^^,^^*b*^	7.59 ± 2.55^*a*^^,^^*b*^	<0.001
	≥65	4.89 ± 1.65	5.85 ± 2.00^*a*^	7.46 ± 2.78^*a*^^,^^*b*^	7.44 ± 2.71^*a*^	<0.001
Types of antidiabetic agents	Insulin and/or sulfonylureas					
	Yes (n = 275)	n = 77	n = 134	n = 46	n = 18	
	No (n = 724)	n = 423	n = 252	n = 39	n = 10	
Average glucose (mmol/L)	Yes	6.85 ± 1.01	8.30 ± 1.26^*a*^	9.91 ± 1.85^*a*^^,^^*b*^	11.86 ± 2.95^*a*^^,^^*b*^^,^^*c*^	<0.001
	No	6.84 ± 1.01	8.18 ± 1.30^*a*^	9.93 ± 1.65^*a*^^,^^*b*^	13.86 ± 3.04^*a*^^,^^*b*^^,^^*c*^	<0.001
SD (mmol/L)	Yes	1.98 ± 0.53^*d*^	2.40 ± 0.58^*a*^^,^^*d*^	2.82 ± 0.71^*a*^^,^^*b*^	2.89 ± 0.78^*a*^^,^^*b*^	<0.001
	No	1.74 ± 0.46	2.05 ± 0.52^*a*^	2.54 ± 0.69^*a*^^,^^*b*^	2.88 ± 0.81^*a*^^,^^*b*^	<0.001
CV (%)	Yes	29.1 ± 6.7^*d*^	29.0 ± 5.6^*d*^	29.2 ± 7.5^*d*^	24.8 ± 5.5^*b*^^,^^*d*^	0.054
	No	25.4 ± 5.3	25.1 ± 5.1	25.6 ± 5.7	20.7 ± 3.0^*a*^^,^^*c*^	0.042
MAGE (mmol/L)	Yes	5.23 ± 1.67^*d*^	6.42 ± 2.20^*a*^^,^^*d*^	7.50 ± 2.38^*a*^^,^^*b*^	7.50 ± 2.84^*a*^	<0.001
	No	4.69 ± 1.56	5.52 ± 1.73^*a*^	6.68 ± 2.25^*a*^^,^^*b*^	7.60 ± 2.09^*a*^^,^^*b*^	<0.001
Renal function	eGFR (mL/min/1.73 m^2^)					
	<60 (n = 227)	n = 114	n = 84	n = 22	n = 7	
	≥60 (n = 772)	n = 386	n = 302	n = 63	n = 21	
Average glucose (mmol/L)	<60	6.61 ± 0.93^*d*^	8.21 ± 1.28^*a*^	9.87 ± 2.21^*a*^^,^^*b*^	12.96 ± 3.58^*a*^^,^^*b*^^,^^*c*^	<0.001
	≥60	6.91 ± 1.02	8.23 ± 1.29^*a*^	9.94 ± 1.58^*a*^^,^^*b*^	12.45 ± 2.98^*a*^^,^^*b*^^,^^*c*^	<0.001
SD (mmol/L)	<60	1.77 ± 0.58	2.20 ± 0.58^*a*^	2.89 ± 1.01^*a*^^,^^*b*^	2.99 ± 0.88^*a*^^,^^*b*^	<0.001
	≥60	1.78 ± 0.45	2.16 ± 0.56^*a*^	2.62 ± 0.56^*a*^^,^^*b*^	2.85 ± 0.76^*a*^^,^^*b*^	<0.001
CV (%)	<60	26.6 ± 7.2	27.0 ± 5.9	29.7 ± 8.6	23.5 ± 5.6	0.137
	≥60	25.8 ± 5.2	26.3 ± 5.5	26.8 ± 6.1	23.3 ± 5.0	0.042
MAGE (mmol/L)	<60	4.71 ± 1.81	5.94 ± 1.91^*a*^	7.62 ± 2.92^*a*^^,^^*b*^	7.79 ± 2.84^*a*^	<0.001
	≥60	4.79 ± 1.52	5.80 ± 1.97^*a*^	6.95 ± 2.10^*a*^^,^^*b*^	7.45 ± 2.53^*a*^^,^^*b*^	<0.001

Data are mean ± SD or median (Q1-Q3). Differences between the 4 HbA1c groups were tested with 1-way analysis of variance and the post hoc Tukey test for normally distributed parameters. Differences between patients stratified by age, types of antidiabetic agents, or renal function in each HbA1c category were tested with Student *t* test or Wilcoxon rank sum test as appropriate.

Abbreviations: CV, coefficient of variation; FLP-CGM, FreeStyle Libre Pro continuous glucose monitoring device; HBGI, high blood glucose index; LBGI, low blood glucose index; MAGE, mean amplitude of glycemic excursions.

^*a*^ *P* < 0.05 vs. HbA1c <  _53_.

^*b*^ *P* < 0.05 vs HbA1c _53-63_.

^*c*^ *P* < 0.05 vs HbA1c _64-74_.

^*d*^ *P* < 0.05 between subgroups.

The prevalence of DR and the severity of DN gradually increased with higher HbA1c (Table 4).

**Table 4.** The prevalence of diabetic retinopathy and the severity of diabetic nephropathy

Parameter	Subgroup	HbA1c _< 53_	HbA1c _53-63_	HbA1c _64-74_	HbA1c _≥ 75_	*P* value
Whole patient population						
Retinopathy (%)		74 (14.8)	105 (27.2)	29 (34.1)	14 (50.0)	<0.001
Normoalbuminuria (%)/ microalbuminuria (%)/ macroalbuminuria (%)		402 (80.4)/75 (15.0)/23 (4.6)	264 (68.4)/96 (24.9)/26 (6.7)	50 (58.8)/24 (28.2)/11 (12.9)	13 (46.4)/8 (28.6)/7 (25.0)	<0.001
Age	Years					
Retinopathy (%)	<65	27 (12.2)	34 (22.5)	12 (27.9)	7 (38.9)	0.001
	≥65	47 (16.9)	71 (30.2)	17 (40.5)	7 (70.0)	<0.001
Normoalbuminuria (%)/ microalbuminuria (%)/ macroalbuminuria (%)	<65	188 (84.7)/26 (11.7)/8 (3.6)	110 (72.8)/31 (20.5)/10 (6.6)	32 (74.4)/8 (18.6)/3 (7.0)^*a*^	9 (50.0)/4 (22.2)/5 (27.8)	0.001
Normoalbuminuria (%)/ microalbuminuria (%)/ macroalbuminuria (%)	≥65	214 (77.0)/49 (17.6)/15 (5.4)	154 (65.5)/65 (27.7)/16 (6.8)	18 (42.9)/16 (38.1)/8 (19.0)	4 (40.0)/4 (40.0)/2 (20.0)	<0.001
Types of antidiabetic agents	Insulin and/or sulfonylureas					
Retinopathy (%)	Yes	19 (24.7)^*a*^	55 (41.0)^*a*^	14 (30.4)	11 (61.1)	0.010
	No	55 (13.0)	50 (19.8)	15 (38.5)	3 (30.0)	<0.001
Normoalbuminuria (%)/ microalbuminuria (%)/ macroalbuminuria (%)	Yes	54 (70.1)/14 (18.2)/9 (11.7)^*a*^	77 (57.5)/43 (32.1)/14 (10.4)^*a*^	26 (56.5)/14 (30.4)/6 (13.0)	9 (50.0)/5 (27.8)/4 (22.2)	0.253
Normoalbuminuria (%)/ microalbuminuria (%)/ macroalbuminuria (%)	No	348 (82.3)/61 (14.4)/14 (3.3)	187 (74.2)/53 (21.0)/12 (4.8)	24 (61.5)/10 (25.6)/5 (12.8)	4 (40.0)/3 (30.0)/3 (30.0)	<0.001
Renal function	eGFR (mL/min/1.73 m^2^)					
Retinopathy (%)	<60	30 (26.3)^*a*^	32 (38.1)^*a*^	10 (45.5)	5 (71.4)	0.023
	≥60	44 (11.4)	73 (24.2)	19 (30.2)	9 (42.9)	<0.001
Normoalbuminuria (%)/ microalbuminuria (%)/ macroalbuminuria (%)	<60	68 (59.6)/31 (27.2)/15 (13.2)^*a*^	39 (46.4)/30 (35.7)/15 (17.9)^*a*^	5 (22.7)/8 (36.4)/9 (40.9)^*a*^	2 (28.6)/2 (28.6)/3 (42.9)	0.006
Normoalbuminuria (%)/ microalbuminuria (%)/ macroalbuminuria (%)	≥60	334 (86.5)/44 (11.4)/8 (2.1)	225 (74.5)/66 (21.9)/11 (3.6)	45 (71.4)/16 (25.4)/2 (3.2)	11 (52.4)/6 (28.6)/4 (19.0)	<0.001

Data are number of patients (%). Differences between the 4 hemoglobin A1c groups were tested with Fisher exact test. Difference between patients stratified by age, types of antidiabetic agents, or renal function in each hemoglobin A1c category were tested with Fisher exact test.

^*a*^ *P* < 0.05 between subgroups.

### Glycemic Variability Across HbA1c Categories According to Age

To investigate whether FLP-CGM–derived metrics differed according to HbA1c, patients were further classified into 8 groups stratified according to HbA1c and age (< 65 years or ≥ 65 years). Patients aged ≥ 65 years with higher HbA1c levels were more likely to have a longer duration of DM and higher triglyceride levels (Supplementary Table 1 (22)). Patients aged < 65 years with higher HbA1c levels were more likely to be younger and to have a longer duration of DM, higher BMI, higher total cholesterol levels, lower high-density lipoprotein cholesterol levels, higher triglyceride levels, and higher eGFR levels. Regardless of age, patients with higher HbA1c levels were more likely to be treated with insulin and oral antidiabetic drugs, including SGLT-2 inhibitors and GLP-1 receptor agonists.

Across the 4 HbA1c categories, the distribution of time spent in different blood glucose ranges is shown in Tables 3 and 5. In patients aged ≥ 65 years, the TBR^ < 3.9 mmol/L^ during both a 24-hour period and daytime was higher in the HbA1c <  _53_ category than in other HbA1c categories. Comparisons between HbA1c subgroups stratified according to age showed that compared with patients aged < 65 years, those aged ≥ 65 years in the HbA1c <  _53_ category had a significantly higher TAR^ > 10 mmol/L^ during daytime, higher TBR^ < 3.9 mmol/L^ during nighttime, higher SD and CV and lower TIR^3.9–10 mmol/L^ during both a 24-hour period and daytime, and lower TAR^ > 10 mmol/L^ during nighttime. In the HbA1c _53-63_ category, patients aged ≥ 65 years had a significantly lower TAR^ > 10 mmol/L^ during nighttime than those aged < 65 years. In the HbA1c _64-74_ category, patients aged ≥ 65 years had a significantly higher TIR^3.9–10 mmol/L^ during nighttime and lower TAR^ > 10 mmol/L^ during nighttime than those aged < 65 years. There were no significant differences between Hb1Ac categories in the percentage of patients who failed to reach the TBR^ < 3.9 mmol/L < ^4% or TBR^ < 3.0 mmol/L^ < 1% criteria (Table 6).

**Table 5.** TIR, TBR, and TAR during a 24-h period, daytime, and nighttime in patients stratified by age

Parameter	Years	HbA1c _< 53_	HbA1c _53-63_	HbA1c _64-74_	HbA1c _≥ 75_	*P* value
	<65 (n = 434)	n = 222	n = 151	n = 43	n = 18	
	≥65 (n = 565)	n = 278	n = 235	n = 42	n = 10	
TIR^3.9–10 mmol/L^ (%)	<65	92.3 (87.2-95.6)^*d*^	80.3 (69.1-87.1)^*a*^	52.9 (36.1-65.8)^*a*^^,^^*b*^	26.7 (6.77-38.4)^*a*^^,^^*b*^^,^^*c*^	<0.001
	≥65	88.5 (82.7-94.7)	76.4 (66.2-86.2)^*a*^	57.4 (41.3-70.6)^*a*^^,^^*b*^	32.9 (9.38-52.5)^*a*^^,^^*b*^^,^^*c*^	<0.001
TBR^ < 3.9 mmol/L^ (%)	<65	0.65 (0.00-2.47)	0.00 (0.00-0.78)^*a*^	0.00 (0.00-0.65)^*a*^	0.00 (0.00-0.00)^*a*^	<0.001
	≥65	0.78 (0.00-3.78)	0.00 (0.00-1.04)^*a*^	0.00 (0.00-1.04)^*a*^	0.00 (0.00-0.00)^*a*^	<0.001
TBR^ < 3.0 mmol/L^ (%)	<65	0.00 (0.00-0.00)	0.00 (0.00-0.00)	0.00 (0.00-0.00)	0.00 (0.00-0.00)	0.042
	≥65	0.00 (0.00-0.13)	0.00 (0.00-0.00)^*a*^	0.00 (0.00-0.00)	0.00 (0.00-0.00)	0.008
TAR^ > 10.0 mmol/L^ (%)	<65	5.21 (2.47-10.9)	18.1 (11.9-30.5)^*a*^	44.8 (32.4-63.9)^*a*^^,^^*b*^	72.8 (61.6-93.2)^*a*^^,^^*b*^^,^^*c*^	<0.001
	≥65	6.58 (2.60-14.2)	22.8 (11.3-32.8)^*a*^	41.9 (28.0-50.2)^*a*^^,^^*b*^	67.1 (47.5-90.6)^*a*^^,^^*b*^^,^^*c*^	<0.001
TAR^ > 13.9 mmol/L^ (%)	<65	0.00 (0.00-0.39)	1.04 (0.00-3.65)^*a*^	8.85 (3.91-21.5)^*a*^^,^^*b*^	26.3 (17.1-60.1)^*a*^^,^^*b*^^,^^*c*^	<0.001
	≥65	0.00 (0.00-0.65)	1.30 (0.00-4.69)^*a*^	9.64 (3.65-16.15)^*a*^^,^^*b*^	21.4 (11.6-41.0)^*a*^^,^^*b*^	<0.001
TIR^3.9–10 mmol/L^ (%), day	<65	91.5 (84.4-95.0)^*d*^	76.9 (63.2-85.1)^*a*^	46.7 (33.3-64.6)^*a*^^,^^*b*^	22.9 (6.3-41.2)^*a*^^,^^*b*^^,^^*c*^	<0.001
	≥65	88.7 (80.6-94.1)	71.9 (58.2-84.4)^*a*^	46.3 (36.7-63.2)^*a*^^,^^*b*^	28.4 (9.7-37.1)^*a*^^,^^*b*^^,^^*c*^	<0.001
TBR^ < 3.9 mmol/L^ (%), day	<65	0.35 (0.00-2.26)	0.00 (0.00-0.52)^*a*^	0.00 (0.00-0.17)^*a*^	0.00 (0.00-0.00)^*a*^	<0.001
	≥65	0.35 (0.00-2.60)	0.00 (0.00-0.52)^*a*^	0.00 (0.00-0.52)^*a*^	0.00 (0.00-0.00)^*a*^	<0.001
TBR^ < 3.0 mmol/L^ (%), day	<65	0.00 (0.00-0.00)	0.00 (0.00-0.00)	0.00 (0.00-0.00)	0.00 (0.00-0.00)	0.258
	≥65	0.00 (0.00-0.00)	0.00 (0.00-0.00)	0.00 (0.00-0.00)	0.00 (0.00-0.00)	0.157
TAR^ > 10.0 mmol/L^ (%), day	<65	6.25 (2.95-13.4)^*d*^	22.1 (13.9-35.9)^*a*^	53.3 (31.8-66.7)^*a*^^,^^*b*^	77.1 (58.9-93.8)^*a*^^,^^*b*^^,^^*c*^	<0.001
	≥65	8.51 (3.47-17.7)	27.4 (14.4-41.8)^*a*^	53.5 (36.8-61.1)^*a*^^,^^*b*^	71.6 (62.9-90.3)^*a*^^,^^*b*^^,^^*c*^	<0.001
TAR^ > 13.9 mmol/L^ (%), day	<65	0.00 (0.00-0.52)	1.39 (0.00-4.17)^*a*^	10.8 (4.17-21.9)^*a*^^,^^*b*^	30.5 (16.9-62.1)^*a*^^,^^*b*^^,^^*c*^	<0.001
	≥65	0.00 (0.00-0.87)	1.56 (0.00-6.25)^*a*^	11.4 (4.49-21.4)^*a*^^,^^*b*^	23.8 (12.2-51.7)^*a*^^,^^*b*^	<0.001
TIR^3.9–10 mmol/L^ (%), night	<65	97.40 (91.7-100.0)	93.2 (81.8-97.9)^*a*^	63.5 (33.3-81.8)^*a*^^,^^*b*^^,^^*d*^	29.7 (6.77-50.5)^*a*^^,^^*b*^^,^^*c*^	<0.001
	≥65	97.4 (88.5-100.0)	94.3 (84.1-99.5)^*a*^	84.1 (66.7-92.7)^*a*^^,^^*b*^	33.9 (9.90-94.8)^*a*^^,^^*b*^	<0.001
TBR^ < 3.9 mmol/L^ (%), night	<65	0.00 (0.00-2.60)^*d*^	0.00 (0.00-0.52)^*a*^	0.00 (0.00-0.52)	0.00 (0.00-0.00)^*a*^	<0.001
	≥65	0.52 (0.00-7.29)	0.00 (0.00-1.04)^*a*^	0.00 (0.00-3.65)	0.00 (0.00-0.00)	<0.001
TBR^ < 3.0 mmol/L^ (%), night	<65	0.00 (0.00-0.00)	0.00 (0.00-0.00)	0.00 (0.00-0.00)	0.00 (0.00-0.00)	0.163
	≥65	0.00 (0.00-0.00)	0.00 (0.00-0.00)	0.00 (0.00-0.00)	0.00 (0.00-0.00)	0.048
TAR^ > 10.0 mmol/L^ (%), night	<65	0.00 (0.00-2.60)^*d*^	5.73 (0.00-15.1)^*a*^^,^^*d*^	33.9 (10.9-60.8)^*a*^^,^^*b*^^,^^*d*^	70.3 (49.5-93.2)^*a*^^,^^*b*^^,^^*c*^	<0.001
	≥65	0.00 (0.00-0.52)	2.08 (0.00-11.5)^*a*^	11.2 (4.17-27.1)^*a*^,*b*	66.2 (5.21-90.1)^*a*^,*b*	<0.001
TAR^ > 13.9 mmol/L^ (%), night	<65	0.00 (0.00-0.00)	0.00 (0.00-0.00)^*a*^	1.04 (0.00-14.06)^*a*^^,^^*b*^	18.5 (5.21-47.4)^*a*^^,^^*b*^^,^^*c*^	<0.001
	≥65	0.00 (0.00-0.00)	0.00 (0.00-0.00)^*a*^	0.00 (0.00-5.21)^*a*^^,^^*b*^	9.11 (0.00-28.7)^*a*^^,^^*b*^	<0.001

Data are median (Q1-Q3). Differences between the 4 hemoglobin A1c groups were tested using the Kruskal-Wallis test with the post hoc Steel-Dwass test. Differences between patients stratified by age in each hemoglobin A1c category were tested with the Wilcoxon rank-sum test.

Abbreviations: TAR, time above range; TBR, time below range; TIR, time in range.

^*a*^ *P* < 0.05 vs. HbA1c <  _53_.

^*b*^ *P* < 0.05 vs HbA1c _53-63_.

^*c*^ *P* < 0.05 vs HbA1c _64-74_.

^*d*^ *P* < 0.05 between subgroups.

**Table 6.** Percentages of patients who failed to reach the TBR criteria

Parameter	Subgroup	HbA1c _< 53_	HbA1c _53-63_	HbA1c _64-74_	HbA1c _≥ 75_	*P* value
Age	Years					
TBR^ < 3.9 mmol/L^ (%) ≥ 4%	<65	42 (18.9)	18 (11.9)	6 (14.0)	0 (0.0)	0.070
	≥65	66 (23.7)	29 (12.3)	5 (11.9)	0 (0.0)	0.002
TBR^ < 3.0 mmol/L^ (%) ≥ 1%	<65	16 (7.2)	13 (8.6)	4 (9.3)	0 (0.0)	0.673
	≥65	22 (7.9)	12 (5.1)	4 (9.5)	0 (0.0)	0.433
Types of antidiabetic agents	Insulin and/or sulfonylureas					
TBR^ < 3.9 mmol/L^ (%) ≥ 4%	Yes	34 (44.2)^*a*^	34 (25.4)^*a*^	10 (21.7)^*a*^	0 (0.0)	<0.001
	No	74 (17.5)	13 (5.2)	1 (2.6)	0 (0.0)	<0.001
TBR^ < 3.0 mmol/L^ (%) ≥ 1%	Yes	19 (24.7)^*a*^	19 (14.2)^*a*^	8 (17.4)^*a*^	0 (0.0)	0.043
	No	19 (4.5)	6 (2.4)	0 (0.0)	0 (0.0)	0.409
Renal function	eGFR (mL/min/1.73 m^2^)					
TBR^ < 3.9 mmol/L^ (%) ≥ 4%	<60	37 (32.5)^*a*^	16 (19.0)^*a*^	4 (18.2)	0 (0.0)	0.059
	≥60	71 (18.4)	31 (10.3)	7 (11.1)	0 (0.0)	0.003
TBR^ < 3.0 mmol/L^ (%) ≥ 1%	<60	12 (10.5)	8 (9.5)	3 (13.6)	0 (0.0)	0.889
	≥60	26 (6.7)	17 (5.6)	5 (7.9)	0 (0.0)	0.642

Data are number of patients (%). Differences between the four HbA1c groups were tested with Fisher’s exact test. Differences between patients stratified by age, types of anti-diabetic agents, or renal function in each HbA1c category were tested with Fisher’s exact test.

Abbreviations: eGFR, estimated glomerular filtration rate; TAR, time above range; TBR, time below range; TIR, time in range.

^*a*^ *P* < 0.05 between subgroups.

In the HbA1c _64-74_ category, there was a significant difference between patients aged ≥ 65 and < 65 years in the severity of DN (Table 4).

### Glycemic Variability Across HbA1c Categories According to Types of Antidiabetic Agents

To further explore whether FLP-CGM–derived metrics differed according to HbA1c, patients were further classified into 8 groups stratified according to HbA1c and types of antidiabetic agents. Regardless of whether patients took insulin and/or sulfonylureas, those with higher HbA1c levels were more likely to be younger and to have higher BMI and triglyceride levels (Supplementary Table 2 (22)). Among patients treated with insulin and/or sulfonylureas, those with higher HbA1c levels were less likely to be treated with sulfonylureas and more likely to be treated with insulin at higher doses. Among patients not treated with insulin and/or sulfonylureas, those with higher HbA1c levels were more likely to be treated with SGLT- 2 inhibitors, thiazolidine, and GLP-1 receptor agonists.

Across the 4 HbA1c categories, the distribution of time spent in different blood glucose ranges is shown in Tables 3 and 7. Regardless of treatment with insulin and/or sulfonylureas, the TBR^ < 3.9 mmol/L^ during a 24-hour period was higher in the HbA1c <  _53_ category than in other HbA1c categories. Comparisons between HbA1c subgroups stratified according to types of antidiabetic agents showed that in the HbA1c <  _53_ category, compared with patients not treated with insulin and/or sulfonylureas, those treated with these agents demonstrated the following: significantly higher TAR^ > 10 mmol/L^ during both a 24-hour period and nighttime; higher TAR^ > 13.9 mmol/L^ during nighttime; higher TBR^ < 3.9 mmol/L^ and TBR^ < 3.0 mmol/L^ during a 24-hour period, daytime, and nighttime; higher SD, CV, and MAGE; and lower TIR^3.9–10 mmol/L^ during a 24-hour period, daytime, and nighttime. In the HbA1c _53-63_ category, compared with patients not treated with insulin and/or sulfonylureas, those treated with these agents demonstrated the following: significantly higher TAR^ > 10 mmol/L^ during a 24-hour period and daytime; higher TBR^ < 3.9 mmol/L^, TBR^ < 3.0 mmol/L^, and TAR^ > 13.9 mmol/L^ during a 24-hour period, daytime, and nighttime; higher SD, CV, and MAGE; and lower TIR^3.9–10 mmol/L^ during a 24-hour period, daytime, and nighttime. In the HbA1c _64-74_ category, patients treated with insulin and/or sulfonylureas had a significantly higher TBR^ < 3.9 mmol/L^ during daytime and TBR^ < 3.0 mmol/L^ during a 24-hour period, daytime, and nighttime and higher CV. In the HbA1c <  _53_, HbA1c _53-63_, and HbA1c _64-74_ categories, the percentage of patients treated with insulin and/or sulfonylureas who failed to reach either the TBR^ < 3.9 mmol/L < ^4% or TBR^ < 3.0 mmol/L^ < 1% criteria was higher than in those not treated with these agents (Table 6).

**Table 7.** TIR, TBR, and TAR during a 24-h period, daytime, and nighttime in patients stratified by types of antidiabetic agents

Parameter	Insulin and/or sulfonylureas	HbA1c _< 53_	HbA1c _53-63_	HbA1c _64-74_	HbA1c _≥ 75_	*P* value
	Yes (n = 275)	n = 77	n = 134	n = 46	n = 18	
	No (n = 724)	n = 423	n = 252	n = 39	n = 10	
TIR^3.9–10 mmol/L^ (%)	Yes	85.0 (77.5-91.7)^*d*^	74.7 (63.7-82.4)^*a*^^,^^*d*^	53.0 (37.0-67.7)^*a*^^,^^*b*^	35.1 (19.3-52.5)^*a*^^,^^*b*^	<0.001
	No	91.2 (84.9-95.4)	82.2 (68.9-88.5)^*a*^	57.4 (36.1-67.6)^*a*^^,^^*b*^	17.2 (4.95-27.2)^*a*^^,^^*b*^^,^^*c*^	<0.001
TBR^ < 3.9 mmol/L^ (%)	Yes	2.86 (0.39-8.33)^*d*^	0.52 (0.00-4.04)^*a*^^,^^*d*^	0.00 (0.00-2.86)^*a*^	0.00 (0.00-0.00)^*a*^^,^^*b*^	<0.001
	No	0.52 (0.00-2.21)	0.00 (0.00-0.26)^*a*^	0.00 (0.00-0.26)^*a*^	0.00 (0.00-0.00)^*a*^	<0.001
TBR^ < 3.0 mmol/L^ (%)	Yes	0.00 (0.00-0.91)^*d*^	0.00 (0.00-0.13)^*a*^^,^^*d*^	0.00 (0.00-0.00)^*d*^	0.00 (0.00-0.00)^*a*^^,^^*b*^	<0.001
	No	0.00 (0.00-0.00)	0.00 (0.00-0.00)^*a*^	0.00 (0.00-0.00)	0.00 (0.00-0.00)	<0.001
TAR^ > 10.0 mmol/L^ (%)	Yes	7.94 (3.65-14.2)^*d*^	23.6 (14.5-33.5)^*a*^^,^^*d*^	44.9 (27.5-61.3)^*a*^^,^^*b*^	64.9 (47.5-80.7)^*a*^^,^^*b*^	<0.001
	No	5.47 (2.34-12.2)	17.2(10.4-31.1)^*a*^	41.2 (32.4-63.9)^*a*^^,^^*b*^	82.8 (72.8-95.1)^*a*^^,^^*b*^^,^^*c*^	<0.001
TAR^ > 13.9 mmol/L^ (%)	Yes	0.00 (0.00-0.91)	2.15 (0.39-6.51)^*a*^^,^^*d*^	12.30 (4.04-19.58)^*a*^^,^^*b*^	18.80 (9.11-48.57)^*a*^^,^^*b*^	<0.001
	No	0.00 (0.00-0.52)	0.91 (0.00-3.13)^*a*^	8.20 (3.30-16.80)^*a*^^,^^*b*^	32.94 (21.35-70.18)^*a*^^,^^*b*^^,^^*c*^	<0.001
TIR^3.9–10 mmol/L^ (%), day	Yes	85.2(77.6-91.5)^*d*^	68.6 (58.8-79.2)^*a*^^,^^*d*^	45.1(37.3-62.7)^*a*^^,^^*b*^	32.4 (18.1-41.5)^*a*^^,^^*b*^	<0.001
	No	91.0 (82.8-94.8)	77.6 (62.0-86.1)^*a*^	47.6 (30.3-67.5)^*a*^^,^^*b*^	15.8 (6.3-28.5)^*a*^^,^^*b*^^,^^*c*^	<0.001
TBR^ < 3.9 mmol/L^ (%), day	Yes	2.60 (0.35-5.90)^*d*^	0.35 (0.00-2.60)^*a*^^,^^*d*^	0.00 (0.00-1.39)^*a*^^,^^*d*^	0.00 (0.00-0.00)^*a*^^,^^*b*^	<0.001
	No	0.20 (0.00-1.74)	0.00 (0.00-0.17)^*a*^	0.00 (0.00-0.00)^*a*^	0.00 (0.00-0.00)^*a*^	<0.001
TBR^ < 3.0 mmol/L^ (%), day	Yes	0.00 (0.00-0.69)^*d*^	0.00 (0.00-0.17)^*a*^^,^^*d*^	0.00 (0.00-0.00)^*a*^^,^^*d*^	0.00 (0.00-0.00)^*a*^	<0.001
	No	0.00 (0.00-0.00)	0.00 (0.00-0.00)^*a*^	0.00 (0.00-0.00)	0.00 (0.00-0.00)	0.010
TAR^ > 10.0 mmol/L^ (%), day	Yes	10.2 (4.34-17.7)	29.3 (16.5-41.2)^*a*^^,^^*d*^	54.3 (35.6-61.1)^*a*^^,^^*b*^	67.6(58.5-80.6)^*a*^^,^^*b*^	<0.001
	No	6.94 (2.95-16.0)	22.1 (13.2-38.0)^*a*^	52.4 (32.1-69.7)^*a*^^,^^*b*^	84.2 (71.5-93.8)^*a*^^,^^*b*^^,^^*c*^	<0.001
TAR^ > 13.9 mmol/L^ (%), day	Yes	0.00 (0.00-1.22)	2.26 (0.52-7.99)^*a*^^,^^*d*^	12.9 (5.38-21.4)^*a*^^,^^*b*^	22.0 (10.1-52.6)^*a*^^,^^*b*^	<0.001
	No	0.00 (0.00-0.69)	1.22 (0.00-3.91)^*a*^	10.8 (4.17-21.5)^*a*^^,^^*b*^	39.2 (23.6-72.9)^*a*^^,^^*b*^^,^^*c*^	<0.001
TIR^3.9–10 mmol/L^ (%), night	Yes	90.6 (77.6-97.4)^*d*^	89.3 (77.1-96.4)^*d*^	72.9 (41.7-89.6)^*a*^^,^^*b*^	40.6 (10.4-91.2)^*a*^^,^^*b*^	<0.001
	No	97.9 (92.2-100.0)	95.3 (86.2-99.5)^*a*^	76.6 (59.9-92.2)^*a*^^,^^*b*^	16.7 (3.13-34.4)^*a*^^,^^*b*^^,^^*c*^	<0.001
TBR^ < 3.9 mmol/L^ (%), night	Yes	2.60 (0.00-13.5)^*d*^	0.00(0.00-5.73)^*a*^^,^^*d*^	0.00 (0.00-6.25)^*a*^	0.00 (0.00-0.00)^*a*^^,^^*b*^	<0.001
	No	0.00 (0.00-3.65)	0.00 (0.00-0.00)^*a*^	0.00 (0.00-0.00)^*a*^	0.00 (0.00-0.00)^*a*^	<0.001
TBR^ < 3.0 mmol/L^ (%), night	Yes	0.00 (0.00-0.52)^*d*^	0.00 (0.00-0.00)^*d*^	0.00 (0.00-0.00)^*d*^	0.00 (0.00-0.00)^*a*^	0.020
	No	0.00 (0.00-0.00)	0.00 (0.00-0.00)^*a*^	0.00 (0.00-0.00)	0.00 (0.00-0.00)	0.002
TAR^ > 10.0 mmol/L^ (%), night	Yes	0.00 (0.00-4.17)^*d*^	3.91 (0.00-15.6)^*a*^	14.8 (6.77-57.3)^*a*^^,^^*b*^	59.4 (8.85-89.6)^*a*^^,^^*b*^	<0.001
	No	0.00 (0.00-1.04)	3.05 (0.00-10.7)^*a*^	23.4 (4.17-40.1)^*a*^^,^^*b*^	83.3 (65.6-96.9)^*a*^^,^^*b*^^,^^*c*^	<0.001
TAR^ > 13.9 mmol/L^ (%), night	Yes	0.00 (0.00-0.00)^*d*^	0.00 (0.00-0.00)^*a*^^,^^*d*^	1.56 (0.00-13.02)^*a*^^,^^*b*^	16.2 (0.00-36.5)^*a*^^,^^*b*^	<0.001
	No	0.00 (00.00-0.00)	0.00 (0.00-0.00)^*a*^	0.00 (0.00-4.17)^*a*^^,^^*b*^	24.5 (8.85-62.0)^*a*^^,^^*b*^^,^^*c*^	<0.001

Data are median (Q1-Q3). Differences between the 4 hemoglobin A1c groups were tested using the Kruskal-Wallis test with the post hoc Steel-Dwass test. Differences between patients stratified by types of antidiabetic agents in each hemoglobin A1c category were tested with the Wilcoxon rank-sum test.

Abbreviations: TAR, time above range; TBR, time below range; TIR, time in range.

^*a*^ *P* < 0.05 vs. HbA1c <  _53_.

^*b*^ *P* < 0.05 vs HbA1c _53-63_.

^*c*^ *P* < 0.05 vs HbA1c _64-74_.

^*d*^ *P* < 0.05 between subgroups.

There were significant differences in the prevalence of DR and the severity of DN between patients with and without insulin and/or sulfonylureas in the HbA1c <  _53_ and HbA1c _53-63_ categories (Table 4).

### Glycemic Variability Across HbA1c Categories According to Renal Function

To more clearly delineate whether FLP-CGM–derived metrics differed according to HbA1c, patients were classified into 8 groups stratified according to HbA1c and renal function (< 60 mL/min/1.73 m^2^ or ≥ 60 mL/min/1.73 m^2^). Among patients with higher HbA1c levels, those with eGFR < 60 were more likely than those with lower HbA1c levels to have a longer duration of DM and higher triglyceride levels. On the other hand, patients with eGFR ≥ 60 with higher HbA1c levels were more likely to be younger, have a longer duration of DM, and to have higher BMI, eGFR, and triglyceride levels (Supplementary Table 3 (22)). In the eGFR < 60 group, patients with higher HbA1c levels were more likely to be treated with SGLT-2 inhibitors, GLP-1 receptor agonists, and insulin than patients with lower HbA1c levels. In the eGFR ≥ 60 group, patients with higher HbA1c levels were more likely to be treated with dipeptidyl peptidase-4 inhibitors, GLP-1 receptor agonists, insulin, and lipid-lowering agents.

The distribution of time spent in different blood glucose ranges across the 4 HbA1c categories is shown in Tables 3 and 8. In patients with eGFR < 60, the TBR^ < 3.9 mmol/L^ and TBR^ < 3.0 mmol/L^ during a 24-hour period were higher in the HbA1c <  _53_ category than in the HbA1c _53-63_ and in the HbA1c ≥  _75_ categories. In patients with eGFR ≥ 60, the TBR^ < 3.9 mmol/L^ during a 24-hour period in the HbA1c <  _53_ category was higher than in the other HbA1c categories. Comparisons between HbA1c subgroups stratified according to renal function showed that in the HbA1c <  _53_ category, patients with eGFR < 60 had a significantly higher TBR^ < 3.9 mmol/L^ during a 24-hour period, daytime, and nighttime, higher TBR^ < 3.0 mmol/L^ during a 24-hour period and daytime, lower TIR^3.9–10 mmol/L^ during nighttime, lower TAR^ > 10 mmol/L^ during a 24-hour period and daytime, and lower average glucose than patients with eGFR ≥ 60. In the HbA1c _53-63_ category, patients with eGFR < 60 had a significantly higher TBR^ < 3.0 mmol/L^ during a 24-hour period and higher TBR^ < 3.9 mmol/L^ during daytime than those with eGFR ≥ 60. In the HbA1c _64-74_ category, patients with eGFR < 60 had a significantly higher TBR^ < 3.9 mmol/L^ during daytime than those with eGFR ≥ 60. In the HbA1c <  _53_ and HbA1c _53-63_ categories, the percentage of patients with eGFR < 60 who failed to the reach the TBR^ < 3.9 mmol/L^ < 4% criterion was higher than in those with eGFR ≥ 60 (Table 6).

**Table 8.** TIR, TBR, and TAR during a 24-hour period, daytime, and nighttime in patients stratified by renal function

Parameter	eGFR (mL/min/1.73 m^2^)	HbA1c _< 53_	HbA1c _53-63_	HbA1c _64-74_	HbA1c _≥ 75_	*P* value
	<60 (n = 227)	n = 114	n = 84	n = 22	n = 7	
	≥60 (n = 772)	n = 386	n = 302	n = 63	n = 21	
TIR^3.9–10 mmol/L^ (%)	<60	90.3 (83.9-95.3)	75.3 (66.2-86.5)^*a*^	54.3 (37.8-62.2)^*a*^^,^^*b*^	36.6 (9.4-41.4)^*a*^^,^^*b*^	<0.001
	≥60	90.8 (84.1-94.9)	79.2(67.2-86.7)^*a*^	55.2(36.1-68.0)^*a*^^,^^*b*^	27.2 (7.2-47.4)^*a*^^,^^*b*^^,^^*c*^	<0.001
TBR^ < 3.9 mmol/L^ (%)	<60	1.69 (0.00-6.12)^*d*^	0.13 (0.00-1.89)^*a*^	0.20 (0.00-2.73)	0.00 (0.00-0.00)^*a*^	<0.001
	≥60	0.52 (0.00-2.47)	0.00 (0.00-0.78)^*a*^	0.00 (0.00-0.65)^*a*^	0.00 (0.00-0.00)^*a*^	<0.001
TBR^ < 3.0 mmol/L^ (%)	<60	0.00 (0.00-0.26)^*d*^	0.00 (0.00-0.00)^*d*^	0.00 (0.00-0.00)	0.00 (0.00-0.00)	0.080
	≥60	0.00 (0.00-0.00)	0.00 (0.00-0.00)^*a*^	0.00 (0.00-0.00)	0.00 (0.00-0.00-)	0.004
TAR^ > 10.0 mmol/L^ (%)	<60	4.69 (1.69-11.3)^*d*^	23.6 (11.5-33.1)^*a*^	42.7(36.1-61.6)^*a*^^,^^*b*^	63.4 (58.6-90.6)^*a*^^,^^*b*^	<0.001
	≥60	6.07 (2.73-12.9)	19.5 (11.5-31.8)^*a*^	44.3 (28.0-63.9)^*a*^^,^^*b*^	72.8 (52.6-92.8)^*a*^^,^^*b*^^,^^*c*^	<0.001
TAR^ > 13.9 mmol/L^ (%)	<60	0.00 (0.00-0.39)	1.30 (0.20-4.62)^*a*^	12.6 (4.56-16.0)^*a*^^,^^*b*^	19.7 (11.6-67.8)^*a*^^,^^*b*^	<0.001
	≥60	0.00 (0.00-0.65)	1.11 (0.00-4.04)^*a*^	8.59 (3.65-20.3)^*a*^^,^^*b*^	24.9(17.3-52.3)^*a*^^,^^*b*^^,^^*c*^	<0.001
TIR^3.9–10 mmol/L^ (%), day	<60	90.6 (81.4-95.4)	70.8 (58.4-83.7)^*a*^	49.1 (36.7-57.5)^*a*^^,^^*b*^	34.9(9.73-41.5)^*a*^^,^^*b*^	<0.001
	≥60	89.9 (81.9-94.4)	75.0 (60.2-84.9)^*a*^	46.2 (33.3-64.6)^*a*^^,^^*b*^	23.6 (6.25-37.1)^*a*^^,^^*b*^^,^^*c*^	<0.001
TBR^ < 3.9 mmol/L^ (%), day	<60	0.87 (0.00-4.34)^*d*^	0.00 (0.00-1.22)^*a*^^,^^*d*^	0.09 (0.00-1.39)^*d*^	0.00 (0.00-0.00)^*a*^	<0.001
	≥60	0.35 (0.00-1.91)	0.00 (0.00-0.52)^*a*^	0.00 (0.00-0.00)^*a*^	0.00 (0.00-0.00)^*a*^	<0.001
TBR^ < 3.0 mmol/L^ (%), day	<60	0.00 (0.00-0.17)^*d*^	0.00 (0.00-0.00)	0.00 (0.00-0.00)	0.00 (0.00-0.00)	0.236
	≥60	0.00 (0.00-0.00)	0.00 (0.00-0.00)	0.00 (0.00-0.00)	0.00 (0.00-0.00)	0.132
TAR^ > 10.0 mmol/L^ (%), day	<60	6.16 (2.26-13.7)^*d*^	27.8 (14.6-41.6)^*a*^	50.7 (42.5-62.6)^*a*^,^*b*^	65.1 (58.5-90.3)^*a*^,^*b*^	<0.001
	≥60	7.81 (3.47-16.5)	23.7 (13.9-39.2)^*a*^	53.8 (32.1-66.7)^*a*^^,^^*b*^	76.4 (62.9-93.8)^*a*^^,^^*b*^^,^^*c*^	<0.001
TAR^ > 13.9 mmol/L^ (%), day	<60	0.00 (0.00-0.52)	1.57 (0.17-6.16)^*a*^	12.9 (5.40-19.1)^*a*^^,^^*b*^	19.8 (10.1-72.7)^*a*^^,^^*b*^	<0.001
	≥60	0.00 (0.00-0.69)	1.39 (0.00-5.21)^*a*^	10.94(4.34-21.9)^*a*^^,^^*b*^	26.6 (20.0-54.0)^*a*^^,^^*b*^^,^^*c*^	<0.001
TIR^3.9–10 mmol/L^ (%), night	<60	95.8 (87.0-100.0)^*d*^	92.2 (80.5-99.0)	73.4 (34.4-83.3)^*a*^^,^^*b*^	29.2 (8.33-78.1)^*a*^^,^^*b*^	<0.001
	≥60	97.9 (91.7-100.0)	94.3 (83.3-99.0)^*a*^	74.0 (46.9-90.6)^*a*^^,^^*b*^	30.2 (9.90-66.2)^*a*^^,^^*b*^^,^^*c*^	<0.001
TBR^ < 3.9 mmol/L^ (%), night	<60	2.60 (0.00-10.4)^*d*^	0.00 (0.00-1.04)^*a*^	0.00 (0.00-9.38)	0.00 (0.00-0.00)^*a*^	<0.001
	≥60	0.00 (0.00-2.60)	0.00 (0.00-0.52)^*a*^	0.00 (0.00-0.52)^*a*^	0.00 (0.00-0.00)^*a*^	<0.001
TBR^ < 3.0 mmol/L^ (%), night	<60	0.00 (0.00-0.00)	0.00 (0.00-0.00)	0.00 (0.00-0.00)	0.00 (0.00-0.00)	0.152
	≥60	0.00 (0.00-0.00)	0.00 (0.00-0.00)	0.00 (0.00-0.00)	0.00 (0.00-0.00)	0.035
TAR^ > 10.0 mmol/L^ (%), night	<60	0.00 (0.00-0.52)	3.91 (0.00-13.0)^*a*^	19.8 (4.69-33.3)^*a*^^,^^*b*^	70.8 (21.9-91.7)^*a*^^,^^*b*^	<0.001
	≥60	0.00 (0.00-1.56)	3.13 (0.00-12.5)^*a*^	18.8 (6.25-53.1)^*a*^^,^^*b*^	69.8 (33.9-90.1)^*a*^^,^^*b*^^,^^*c*^	<0.001
TAR^ > 13.9 mmol/L^ (%), night	<60	0.00 (0.00-0.00)	0.00 (0.00-0.00)^*a*^	0.52 (0.00-14.1)^*a*^^,^^*b*^	17.7 (2.60-53.1)^*a*^^,^^*b*^	<0.001
	≥60	0.00 (0.00-0.00)	0.00 (0.00-0.00)^*a*^	0.52 (0.00-6.77)^*a*^^,^^*b*^	16.2 (2.60-47.4)^*a*^^,^^*b*^^,^^*c*^	<0.001

Data are median (Q1-Q3). Differences between the 4 hemoglobin A1c groups were tested using the Kruskal-Wallis test with the post hoc Steel-Dwass test. Differences between patients stratified by renal function in each hemoglobin A1c category were tested with the Wilcoxon rank-sum test.

Abbreviations: eGFR, estimated glomerular filtration rate; TAR, time above range; TBR, time below range; TIR, time in range.

^*a*^ *P* < 0.05 vs. HbA1c <  _53_.

^*b*^ *P* < 0.05 vs HbA1c _53-63_.

^*c*^ *P* < 0.05 vs HbA1c _64-74_.

^*d*^ *P* < 0.05 between subgroups.

There were significant differences in the prevalence of DR between patients with eGFR ≥ 60 and eGFR < 60 in the HbA1c <  _53_ and HbA1c _53-63_ categories, as well as in the severity of DN between patients in the HbA1c <  _53_, HbA1c _53-63_, and HbA1c _64-74_ categories (Table 4).

## Discussion

In this study, we clarified the characteristics of patients who might derive clinical benefit from the use of CGM metrics to complement HbA1c monitoring. The results newly showed that in terms of evaluating glucose variability and hypoglycemic episodes, using CGM metrics to complement HbA1c monitoring would be beneficial for patients treated with insulin and/or sulfonylureas, older patients, and patients with CKD (Supplementary Table 4 (22)). These data should provide useful clues and thereby facilitate more individualized glycemic management.

In general, insulin and sulfonylureas have been reported to increase the risk of hypoglycemic episodes. According to a survey of treatment-related severe hypoglycemia conducted by the JDS, more than 90% of Japanese patients with T2DM who experienced severe hypoglycemia were being treated with insulin and/or sulfonylureas (23). Similarly, our study demonstrated that patients treated with insulin and/or sulfonylureas had a higher TBR compared with those not treated with these agents. In particular, patients in the HbA1c <  _53_ category who were treated with these agents had a higher TBR^ < 3.9 mmol/L^ than those in other HbA1c categories. Patients in the HbA1c <  _53_ and HbA1c _53-63_ categories who received these agents had a higher TBR than those who did not. Even patients in the HbA1c _64-74_ category who were treated with these agents had a higher TBR^ < 3.0 mmol/L^ than those who were not, and about 20% of those who received these drugs failed to reach each TBR criterion. Similarly, a very recent study showed that the frequency of nocturnal TBR episodes was not lower in insulin-treated T2DM patients with HbA1c ≥ 63 mmol/mol compared with those in lower HbA1c categories (24). These data suggest that higher HbA1c levels do not always protect against the occurrence of hypoglycemic episodes. Thus, physicians need to take measures to prevent hypoglycemic episodes in patients on insulin and/or sulfonylureas in the clinical setting, regardless of HbA1c levels.

Intriguingly, there were differences in glucose variability between patients treated with or without insulin and/or sulfonylureas in the HbA1c <  _53_ and HbA1c _53-63_ categories because patients treated with these agents had a significantly lower TIR as well as a higher TAR and TBR than their counterparts. Similar findings were observed for SD, CV, and MAGE. These data suggest that even within the same HbA1c category, the quality of glycemic control differs depending on whether patients receive insulin and/or sulfonylureas. In this regard, it is notable that there were moderate differences in the prevalence of DR and the severity of DN between patients treated with and without insulin and/or sulfonylureas in the HbA1c <  _53_ and HbA1c _53-63_ categories. However, this was a cross-sectional study, and the use of insulin and/or sulfonylureas might only be a marker of advanced T2DM. Further studies are needed to clarify whether differences in glucose variability despite similar HbA1c levels are associated with long-term outcomes.

CKD can cause alterations in glucose and insulin metabolism in patients with diabetes (25). However, impairments in renal gluconeogenesis, insulin clearance and degradation, and counterregulatory hormone responses may cause hypoglycemic episodes in T2DM patients with CKD (25). Indeed, a cohort study demonstrated that across various degrees of hypoglycemia severity, the rate of hypoglycemia in T2DM patients was higher in those with CKD than in those without (26). Consistent with this finding, in the HbA1c <  _53_ category in this study, patients with eGFR < 60 had a higher TBR than those with eGFR ≥ 60, indicating a difference in the frequency of hypoglycemic episodes. Also, in the HbA1c _53-63_ and the HbA1c _64-74_ categories, patients with eGFR < 60 had a higher TBR than those with eGFR ≥ 60. Furthermore, reduced clearance of antidiabetic agents such as insulin and/or sulfonylureas can cause hypoglycemic episodes in T2DM patients with CKD. In fact, in the HbA1c <  _53_ and HbA1c _53-63_ categories, patients with eGFR < 60 and those treated with insulin and/or sulfonylureas had the highest TBR (Supplementary Table 5 (22)). However, HbA1c alone does not provide such information. In addition, a previous study demonstrated that in the general population, worse renal function (eGFR < 60) was strongly correlated with a higher prevalence of anemia (27). Thus, the emergence of CGM offers more effective ways of monitoring and making therapeutic adjustments, which together allow for fine-tuning of glycemic management in T2DM patients with CKD to avoid hypoglycemic episodes regardless of HbA1c levels.

The JDS and Japan Geriatric Society recommend that HbA1c targets in older people with T2DM should be set based on the presence or absence of medications that have the potential to cause severe hypoglycemia, and the groups classified this population into 3 categories according to cognitive function and activities of daily living (8). These guidelines aim to relax glycemic goals by setting higher HbA1c levels to reduce the risk of hypoglycemia. In the current study, however, 35.3% of older patients did not achieve the TBR^ < 3.9 mmol/L^ target of < 1% that is used in older people, even though diabetes specialists provided treatment while keeping the guidelines in mind. Notably, the risk of hypoglycemia increases with the efficacy of glycemic control as evaluated by HbA1c levels. Our study demonstrated that in the HbA1c <  _53_ category, patients aged ≥ 65 years had a higher TBR^ < 3.9 mmol/L^ during nighttime than those aged < 65 years. A previous study found that older patients had a reduced ability to perceive neuroglycopenic and autonomic hypoglycemic symptoms than middle-aged patients (28). Thus, unrecognized hypoglycemia in older people may be associated with HbA1c < 53 mmol/mol. Taken together, we need to take particular care to prevent the occurrence of hypoglycemia, especially during nighttime, in older patients with lower HbA1c levels. On the other hand, there were no differences in TBR between patients aged ≥ 65 and < 65 years in the HbA1c _53-63_, HbA1c _64-74_, and HbA1c ≥  _75_ categories. However, because unrecognized hypoglycemia is more common in older people, as discussed previously, higher HbA1c may not necessarily protect against hypoglycemia in this population. Thus, assessing the occurrence of hypoglycemic episodes with CGM at least once may be useful for older people, even those with higher HbA1c.

The strengths of this study included its relatively large sample size and multicenter study design. However, the study had several limitations. First, it used a cross-sectional design with drawbacks similar to all analyses of this type. Second, some of the unobserved differences between patients, in particular those in the HbA1c _64-74_ and HbA1c ≥  _75_ categories, might largely have been due to the low number of patients. The frequency of hypoglycemic episodes may have differed between patients in the HbA1c _64-74_ and HbA1c ≥  _75_ categories because patients in the HbA1c ≥  _75_ category had much higher TAR and much lower TIR and TBR than those in other categories. In addition, although all individuals in the HbA1c ≥  _75_ category met the TBR^ < 3.9 mmol/L^ < 4% and TBR^ < 3.0 mmol/L^ < 1% criteria, some in HbA1c _64-74_ category did not. Third, stratification was not performed according to other potential patient and disease factors that are known to impact glucose variability and the frequency of hypoglycemia. Fourth, FLP-CGM–derived metrics were evaluated based on FLP-CGM measurements obtained during a limited period. Thus, these metrics may not represent the subjects’ overall glycemic control. To most accurately assess glucose variability with FLP-CGM at baseline, we only recruited patients with stable control. In addition, we used a blind CGM system that prevented subjects from altering their lifestyle behaviors based on the results of glucose readings. Fifth, we only recruited Japanese patients with T2DM and without a history of cardiovascular events. Sixth, the study data were obtained from 8 consecutive days of CGM, excluding the first 2 days and the last 2 days, which have been reported to be inaccurate. Thus, the FLP-CGM–derived metrics may not accurately represent the overall glycemic control. These constraints may limit the generalizability of our results. Seventh, we used serum creatinine to estimate the glomerular filtration rate. However, eGFR is affected by several factors, including the secretion rate of creatinine in the tubules and the metabolism of creatinine from creatine in the muscles (29). Thus, eGFR calculation is less accurate in older people because muscle mass decreases with age (29). Finally, CKD was defined by a single measurement of eGFR < 60 mL/min/1.73 m^2^ rather than by several measurements performed at intervals of at least 3 months (30). In addition, the low number of participants, especially those with more advanced stages of CKD, limited our statistical power to perform further stratified analyses (ie, eGFR ≥ 90, 60-89, 30-59, 15-29, and < 15 mL/min/1.73 m^2^).

In conclusion, we demonstrated that higher HbA1c levels do not always protect against the occurrence of hypoglycemic episodes, and patients within the same HbA1c category may carry different risk. In clinical practice, however, individualized target HbA1c levels are largely adopted without evaluating the quality of glycemic control. In terms of evaluating glucose variability and hypoglycemic episodes, using CGM metrics to complement HbA1c monitoring is beneficial in patients treated with insulin and/or sulfonylureas, older patients, and patients with CKD.

## Data Availability

Some or all datasets generated during and/or analyzed during the current study are not publicly available but are available from the corresponding author on reasonable request.

## Abbreviations

ADA: American Diabetes Association
BMI: body mass index
CGM: continuous glucose monitoring
CKD: chronic kidney disease
CV: coefficient of variation
DN: diabetic nephropathy
DR: diabetic retinopathy
eGFR: estimated glomerular filtration rate
FLP: FreeStyle Libre Pro
GLP-1: glucagon-like peptide-1
HbA1c: hemoglobulin A1c
JDS: Japan Diabetes Society
MAGE: mean amplitude of glycemic excursion
Q: quartile
SGLT-2: sodium-glucose cotransporter 2
T2DM: type 2 diabetes mellitus
TAR: time above range
TBR: time below range
TIR: time in range

## Clinical Trial Information

The study was registered in the University Hospital Medical Information Network Clinical Trials Registry (UMIN000032325).
